# The Long Term Residual Effects of COVID-Associated Coagulopathy

**DOI:** 10.3390/ijms24065514

**Published:** 2023-03-14

**Authors:** Marco Ranucci, Ekaterina Baryshnikova, Martina Anguissola, Sara Pugliese, Mara Falco, Lorenzo Menicanti

**Affiliations:** 1 Department of Cardiovascular Anesthesia and Intensive Care, IRCCS Policlinico San Donato, 20097 Milan, Italy; 2 Department of Radiology, Koelliker Hospital, 10134 Turin, Italy; 3 Scientific Directorate, IRCCS Policlinico San Donato, 20097 Milan, Italy

**Keywords:** COVID-19, post-acute COVID-19 syndrome, coagulopathy, fibrinolysis, thrombosis

## Abstract

During the acute phase of COVID-19, many patients experience a complex coagulopathy characterized by a procoagulant pattern. The present study investigates the persistence of hemostatic changes in post-COVID patients at a long-term follow up, and the link with the persistence of physical and neuropsychological symptoms. We completed a prospective cohort study on 102 post-COVID patients. Standard coagulation and viscoelastic tests were performed, along with an assessment of persistent symptoms and recording of acute phase details. A procoagulant state was adjudicated in the presence of fibrinogen > 400 mg/dL, or D-dimer > 500 ng/mL, or platelet count > 450,000 cells/µL, or a maxim clot lysis at viscoelastic test < 2%. A procoagulant state was identified in 75% of the patients at 3 months follow up, 50% at 6 months, and 30% at 12–18 months. Factors associated with the persistence of a procoagulant state were age, severity of the acute phase, and persistence of symptoms. Patients with major physical symptoms carry a procoagulant state relative risk of 2.8 (95% confidence interval 1.17–6.7, *p* = 0.019). The association between persistent symptoms and a procoagulant state raises the hypothesis that an ongoing process of thrombi formation and/or persistent microthrombosis may be responsible for the main physical symptoms in long-COVID patients.

## 1. Introduction

The COVID-19 associated coagulopathy (CoAC) is a complex syndrome complicating the acute phase of COVID-19 infection, first recognized at the beginning of the pandemic in Italy through the use of standard coagulation tests, point-of-care viscoelastic tests (VET), and a biochemical measure of markers of thrombin generation, fibrin generation, platelet activation, and fibrinolysis [1,2,3].

Recognized patterns of CoAC are increased thrombin generation [4,5,6], thrombocytosis in the early phases and thrombocytopenia in late severe conditions [7,8], blunted fibrinolysis [6,9], and high levels of D-dimer [10,11,12].

This complex pattern clearly shows a dynamic behavior, where phases of activation are followed by phases of coagulation factor consumption, exhaustion of the hemostatic system, and, in more severe cases, disseminated intravascular coagulopathy. The main clinical consequences of CoAC are thromboembolic complications, most frequently represented by pulmonary embolism [13,14]. However, thrombi formation may be observed practically everywhere, even in minor, subclinical manifestations of CoAC [15,16]. Extensive imaging analyses clearly confirm the presence of micro and macro thrombotic formation in different organs [17].

Whether this pattern and/or the consequences of CoAC resist and leave a long-term signature in the hemostatic system after the closure of the acute phase of the disease is still not well defined. There is an Indian study with a 3-month and a 6-month follow up where elevated D-dimer values were associated with persistence of symptoms [18]; however, prolonged elevation of D-dimer levels seems not to be associated with the severity of acute phase response [19]. The only study assessing the hemostatic profile after 1 year from the acute phase shows the persistence of high D-dimer and factor VIII levels in 18% and 49% of the patients, respectively, with an increased thrombin generation [20]. 

Since the first reports of hypercoagulability during the acute phase of COVID-19 were based on point-of-care viscoelastic testing (VET), it is reasonable to use this approach to investigate the long-term residual effects of the disease on the hemostatic system. The present study aims to investigate the hemostatic pattern of COVID-19 patients at a long-term follow up, and to assess the association between patterns of the acute phase, persistence of clinical symptoms, and hemostatic system profile.

## 2. Results

The general characteristics of the patient population during the acute phase and at follow-up are shown in Table 1. The hemostatic profile at follow-up is shown in Table 2, separately for patients with or without persistent major physical symptoms (MPS) and/or major neuropsychological symptoms (MNS). During the acute phase, a procoagulant pattern was identified in 99 (97.1%) of the patients, with one or more of the following conditions: peak fibrinogen levels > 400 mg/dL in 99 (97.1%) patients; peak D-Dimer > 500 ng/mL in 87 (84.8%) patients; peak platelet count > 450,000 cells/µL in 17 (15.8%) patients and nadir antithrombin (AT) activity < 70% in 6 (5.9%) patients. After discharge from the hospital, a procoagulant state was still present at follow-up in 38 (37.3%), presenting one or more of the following conditions: fibrinogen levels > 400 mg/dL in 7 (6.9%) patients; D-Dimer > 500 ng/mL in 28 (27.4%) patients; platelet count > 450,000 cells/µL in 17 (15.8%) patients, AT activity < 70% in 4 (3.9%) patients, and ClotPro EXtest maximum lysis (ML) < 2% in 7 (6.9%) patients.

**Figure 1:**
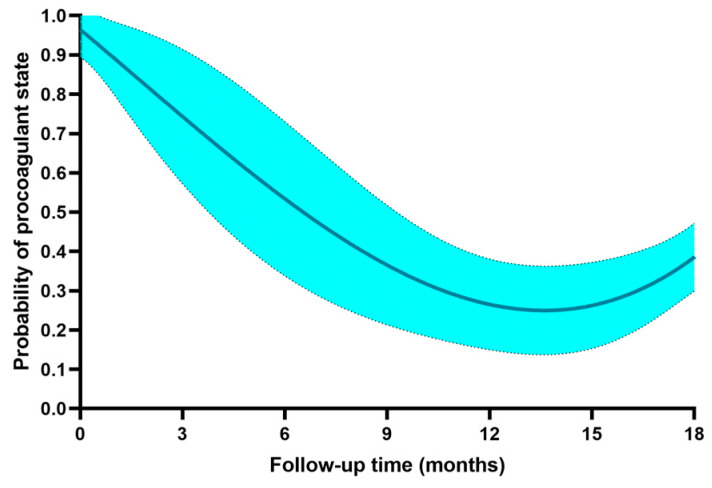
Probability of the persistence of a procoagulant state. Cubic regression function with 95% confidence interval bands.

**Figure 2:**
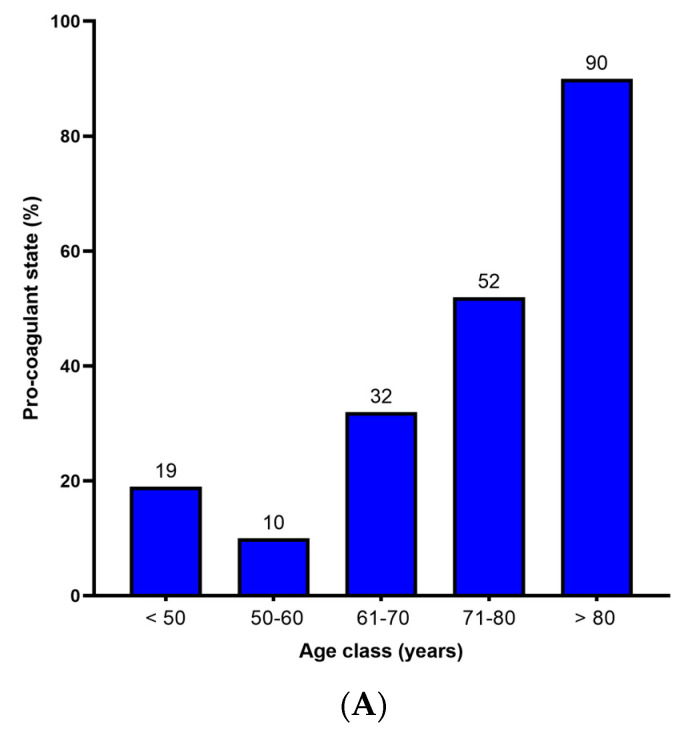

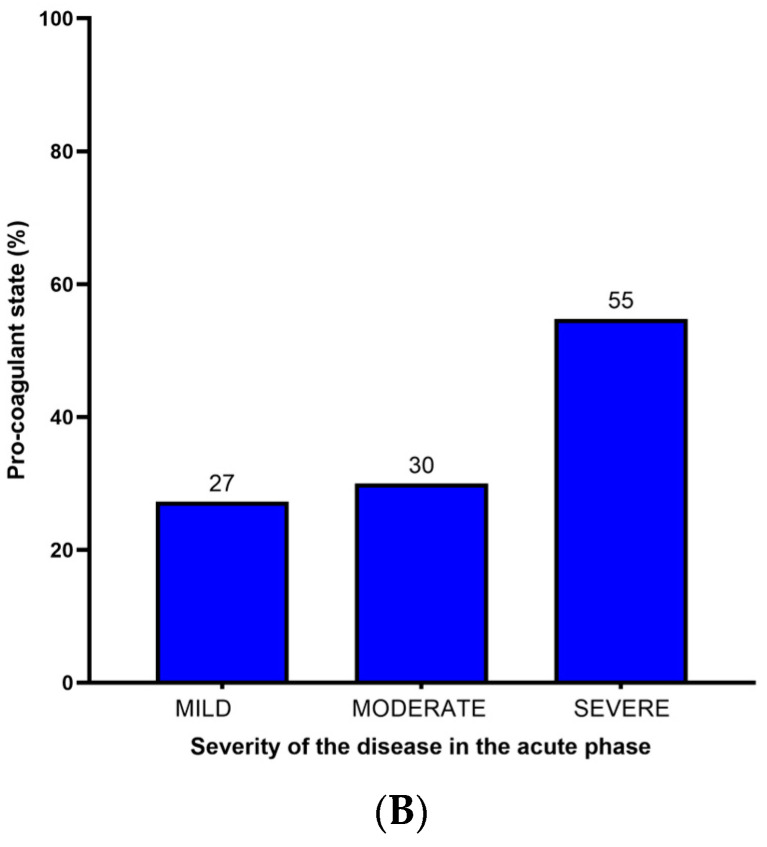
Rate of procoagulant state persistence based on the age at acute phase stage (Panel (**A**)) and severity of the acute phase disease (Panel (**B**)).

The probability of a persistent procoagulant state during follow up is shown in Figure 1 (cubic regression analysis, R^2^ 0.415, *p* = 0.001). Overall, a persistent procoagulant state was still present in 75% of the patient population at 3 months, 50% at 6 months, and 25% at 12 months of follow-up, with a slight increase to 35% at 18 months. The R^2^ value justifies 41% of the follow-up time based relationship, and there are certainly other factors affecting the persistence of a procoagulant state. Among these, persistence of MPS carries a higher rate of a procoagulant state (46.7% vs. 23.8%, relative risk 2.8, 95% confidence interval 1.17–6.7, *p* = 0.019), whereas persistence of MNS did not (40.9% vs. 34.5%). Among the acute phase factors, only age class and the severity of the disease were significantly associated with a procoagulant state at follow up (Figure 2). For increasing age higher than 50 years, there is a significant (*p* = 0.001) incremental increase of the procoagulant state rate, up to 90% in elderly people (> 80 years), and the severity of the disease carries a significant (*p* = 0.05) impact on the procoagulant state rate, especially for patients with a severe pattern that show a risk of procoagulant state at follow up that is almost double the risk for mild to moderate patterns of the disease in the acute phase.

**Figure 3:**
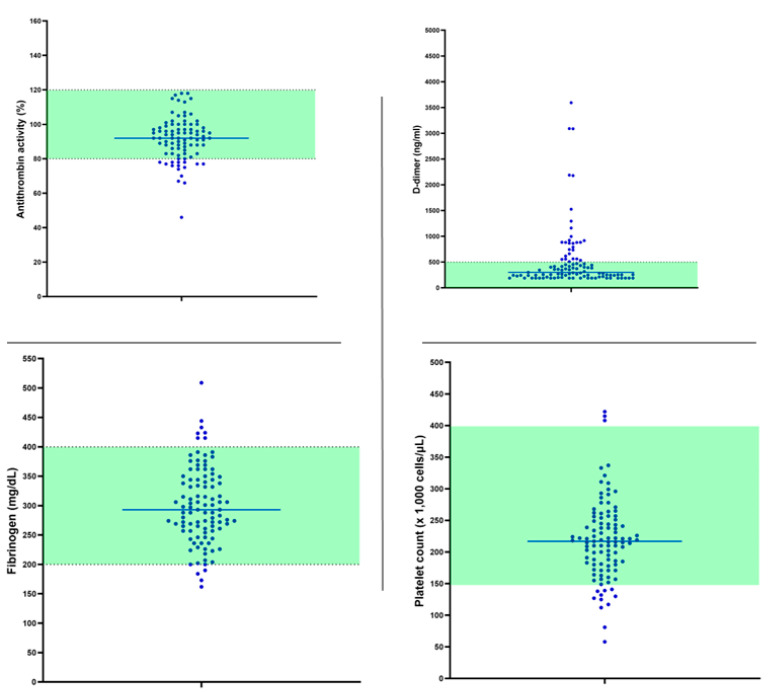
Individual and mean standard coagulation data at follow up. Green area is the normality range.

**Figure 4:**
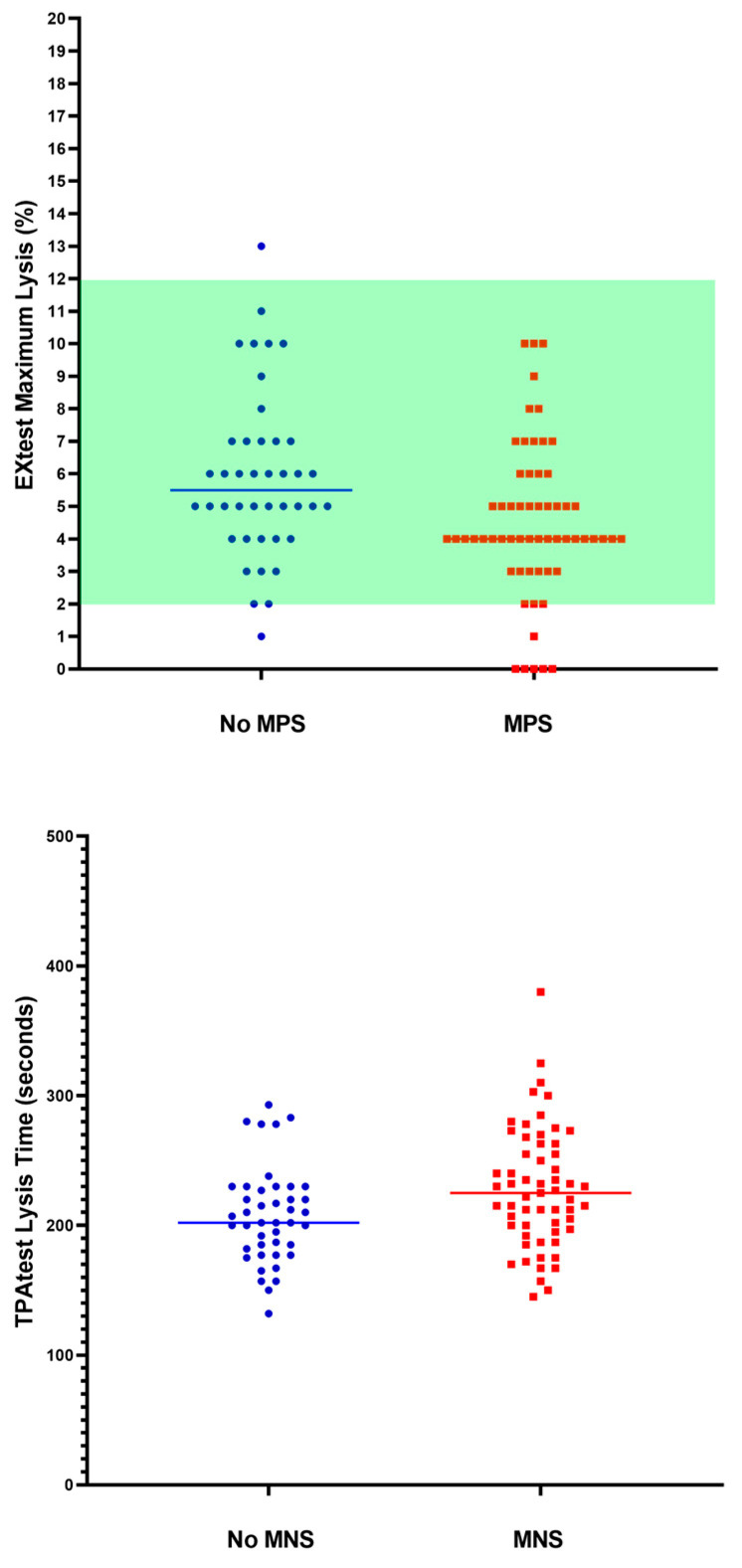
Individual and mean values of fibrinolysis according to the presence of major physical symptoms (MPS) and major neurological symptoms (MNS). Green area is the normality range. No defined normal range exists for TPAtest.

Table 2 reports the hemostatic profile of the patients at follow up, according to the presence of persistent MPS or MNS. Patients with MPS had significantly (*p* = 0.021) higher levels of fibrinogen and of MCF (maximum clot firmness) at the INtest (*p* = 0.026), with a reduced ML (maximum lysis) at both the EXtest (*p* = 0.005) and the INtest (*p* = 0.006), and a longer lysis time at the TPAtest (*p* = 0.033). Patients with MNS had a significantly longer lysis time at the TPAtest (*p* = 0.012). Overall, the general pattern of patients with persistent symptoms is representative of an increased clot firmness, mainly due to fibrinogen contribution, with an impaired fibrinolysis. D-Dimer levels were not different between patients with or without persistent symptoms. Overall, individual and mean standard coagulation data of the patient population are reported in Figure 3. The most evident finding is the presence of high levels of D-dimer in about 30% of the patients. Figure 4 reports the main differences between patients with or without persistent symptoms with respect to VET. For both persistent MPS and MNS, there is evidence of a decreased fibrinolysis with respect to asymptomatic patients.

A sensitivity analysis was conducted on patients with (*n* = 44) or without (*n* = 58) persistent dyspnea. Patients with persistent dyspnea had significantly lower values of EXtest ML (4.4% ± 2.3 vs. 5.6% ± 2.6, mean difference 1.2, 95% confidence interval 0.22 to 2.2, *p* = 0.017) and of INtest ML (3.9% ± 2.1 vs. 5.3% ± 2.6, mean difference 1.42, 95% confidence interval 0.47 to 2.38, *p* = 0.004). The severity of the disease in the acute phase did not affect the fibrinolysis at the EXtest ML (5.3 % ± 2.3 for non-severe cases vs. 4.5% ± 2.9% for severe cases, *p* = 0.172) but affected the fibrinolysis at the INtest ML ((5 % ± 2.4 for non-severe cases vs. 3.9% ± 2.6% for severe cases, *p* = 0.033). When corrected for the severity of the disease, persistent dyspnea remained independently associated with a reduced fibrinolysis both at the EXtest ML (*p* = 0.028) and at the INtest ML (*p* = 0.002).

## 3. Discussion

The main results of our study are (i) persistent hemostatic changes are detectable in 37% of patients hospitalized for COVID-19 at a median follow-up of 17 months; (ii) the main pattern is suggestive of an ongoing fibrinolytic process; and (iii) patients with residual MPS (especially persistent dyspnea) and MNS have significantly lower levels of fibrinolysis. The severity of the disease partially affects the fibrinolysis shutdown.

Much is known about the procoagulant profile of COVID-19 patients during the acute phase of the disease. Much less is known about the short- and long-term sequelae of this pattern. Persistent organ dysfunction, even after several months from the acute phase, has already been observed, and this is a reasonable finding, considering that organs directly (lung) or indirectly (heart, brain, kidney) and severely damaged by the disease take time to recover, and may even not recover at all. Conversely, the hemostatic system is a dynamic structure comprising proteases, glycoproteins, proteins, and cells. All these components are continuously re-synthesized by the liver and the bone marrow, so once the acute insult represented by the inflammatory reaction to virus or bacterial sepsis is overcome, it could be logical to assume that the system comes back to normal, unless in the presence of an ongoing or unresolved process elsewhere in the human body.

Actually, the information on the coagulation profile in long-COVID patients is scarce and mainly limited to short-term follow up. Townsend and associates [19] studied 150 COVID-19 patients at a median of 80 days from the diagnosis, both hospitalized and outpatients. The mean D-Dimer was 327 ng/mL (lower than in our series), with 25% of the patients showing abnormally elevated values (27% in our series). Elevation of D-Dimer was more frequent in older patients and in those with severe acute disease. Our results are basically in agreement with these findings, and the difference in mean D-Dimer values is probably to be ascribed to the different patient population (100% hospitalized in our series vs. 55% in Townsend’s series). Of notice, our follow-up time was 6-fold longer, thus demonstrating that elevated D-Dimers persist over time. Kalaivani et al. [18] checked the D-Dimer levels after 3 and 6 months from the acute phase. After 3 months, 42% of the patients had elevated (>500 ng/mL) D-Dimer levels, with a decrease to 32% at 6 months. These results, again, confirm our finding both in terms of the incidence and the persistence of elevated D-Dimer levels. These rates were found in a population of patients with long-COVID symptoms, and are in-line with our rates of the procoagulant pattern in patients with persistent MPS. A more sound analysis of the coagulation profile at a mean follow up of 12 months after the acute phase was offered by Fan et al. [20], but, unfortunately, in a small series of 39 patients, of whom only 9 suffered a severe pattern of the disease. When compared to a control group, these patients showed significantly higher D-Dimer levels and Factor VIII activity, and a significantly lower AT activity. Thrombin generation and markers of endotheliopathy were significantly higher in long-COVID patients. An increased thrombin generation and endothelial cell activation is confirmed at a 2-month follow up by the study of Gerotfiazas et al. [21], and by von Meijenfeldt et al. [22] at a 4-month follow up in a series of 29 patients, which found an increased thrombin generation and an inhibition of fibrinolysis induced by high levels of plasmin activator inhibitor. Finally, increased levels of antiplasmin were identified in long-COVID patients by Pretorius et al. [23].

Overall, the combined information coming from both our and previous studies is suggestive of persistent or even ongoing micro-thrombi formation and fibrinolysis. This pattern may derive from (i) a continuous process of fibrinolysis of thrombi formed during the acute phase and (ii) an ongoing thrombi formation triggered by endothelial dysfunction and thrombin generation. Of notice, in both our and previous studies, this pattern is more frequent in elderly patients, in those with severe patterns of the disease during the acute phase, and in those with a persistence of major physical symptoms. These results stress the role of the patient-related procoagulant state (a common feature in elderly patients) as well as disease-related procoagulant conditions (more pronounced in severe states during the acute phase). This complex pattern includes an apparent paradox: high levels of fibrin degradation products (D-Dimer) and a concomitant blunting of fibrinolysis. This condition, which was observed even during the acute phase of the disease, has been interpreted in terms of a fibrinolytic process that is present but inadequate to completely counteract the overwhelming amount of fibrin generated by the thrombin burst [2]. Nielsen et al. [24] proposed a very sound and interesting theory to explain the fibrinolysis paradox in COVID-19 patients. They hypothesized that, since the lungs are the primary location of fibrin breakdown and the main source of the D-dimer found in the systemic circulation, hyperfibrinolysis can occur in the pulmonary extra- and intravascular compartments while a systemic hypofibrinolytic state co-exists.

There are many studies addressing thrombotic complications during the acute phase of COVID-19. Tamayo-Velasco et al. retrospectively investigated 2894 patients in the Spanish territory, detecting a rate of major thromboembolic events reaching 3.5%, with a higher associated morbidity and mortality [25]. In an interesting propensity-matched study, De Vita and associates compared thromboembolic events in COVID-19 versus other kinds of infectious respiratory diseases [26]. Before adjustment for the confounders, the thromboembolic event rate was significantly (*p* = 0.001) higher in non-COVID-19 patients (6.9%) than in COVID-19 patients (4.7%); however, after propensity matching, this difference lost significance.

How this long-lasting condition may evolve into clinically relevant effects (namely thrombotic or thromboembolic events) remains an unsolved issue.

Follow-up studies of COVID-19 patients have addressed the incidence of deep vein thrombosis and pulmonary embolism. In a large nationwide study conducted in Sweden [27], patients who tested positive for SARS-CoV-2 were matched to tested negative and were followed for 180 days after the acute phase. The authors found that there was a significant increased incidence rate of deep venous thrombosis and pulmonary embolism, and that the risk of thromboembolic events was higher in patients who experienced a severe pattern of COVID-19. In a series of 83 patients with persistent respiratory symptoms after 2 months from the acute phase, Iqbal et al. [28] found a pulmonary embolism rate of 12.5%. Indirect signs of pulmonary vessel microthrombosis were investigated in a wide series of 767 patients studied 3 months after the acute phase [29]. Impaired lung diffusion was found in 17% of the patient population, and many case reports of pulmonary embolism were reported in long-COVID patients [30]. Pulmonary angiogram in long-COVID patients has been suggested following a specific algorithm based on persistence of respiratory symptoms, lung diffusion tests, and perfusion imaging [31]. The hypothesis that lung vasculature could be the main site of thrombus formation or deposition is supported, in our series, by the finding of significantly higher levels of D-Dimer in patients with persistent dyspnea.

In conclusion, the hemostatic system continues to react to the residual effects of the COVID-19 in a considerable amount of patients, even several months after the acute phase. Unfortunately, there are no studies linking the finding of a long-term procoagulant pattern to overt clinical manifestations. This kind of study would require patient populations larger than the one in our and previously published series, but could provide important information to trigger pre-emptive pharmacological strategies in patients with a long-lasting procoagulant profile.

## 4. Materials and Methods

This is a single-center, prospective cohort study conducted at the IRCCS San Donato, a Clinical Research Hospital partially funded by the Italian Ministry of Health. The Local Ethics Committee (San Raffaele Hospital) approved the experimental design on March 9, 2022, registry number 28/INT/2022. All the patients gave written informed consent. The study has been financed by a grant from the Italian Ministry of Health, within the research projects of the Cardiac Network of the Italian IRCCS (Clinical Research Hospitals). The primary endpoint of this study was the identification of the hemostatic system profile and its link with the acute phase pattern and the persistence of major physical and neuropsychological symptoms from 3 all the way up to 12–18 months from hospital discharge.

### 4.1. Patient Population and Study Procedures

The patients were recruited through a first telephone contact, and those who were reachable and agreed to participate received a date for the study procedure at our hospital. The eligible patient population was represented by subjects hospitalized at our Institution with a diagnosis of COVID-19 infection between 1 January 2021 and 31 July 2022. The planned patient population was 100 patients. The final patient population comprised 102 subjects.

### 4.2. Data Collection and Definitions

Data collection was based on (i) retrieval of the relevant data from the original patient’s files, (ii) a personal interview conducted in a hospital office by dedicated biologists and medical doctors, and (iii) the coagulation parameters measured through standard laboratory tests and viscoelastic tests.

The following items regarding the acute phase hospitalization were collected: demographics (with age classes ≤ 50 years, 51–60 years, 61–70 years, 71–80 years, and > 80 years); disease severity (mild: no oxygen therapy; moderate: nasal oxygen or oxygen mask; severe: non-invasive or invasive ventilation), hospital stay, unit of admission, vaccination (2 doses) at the time of hospital admission; co-morbidities (obesity, hypertension, diabetes, history of coronary disease, heart failure, atrial fibrillation, chronic obstructive pulmonary disease, asthma, active cancer, chronic kidney failure, chronic liver failure, previous cerebrovascular accident, anxiety, depression); and therapy at the time of hospitalization; laboratory exams (peak fibrinogen levels, peak D-Dimer, peak platelet count, nadir platelet count, nadir antithrombin).

Follow-up items included: follow-up duration; any symptom after discharge; work capacity reduced; fatigue, fever, cough, or dyspnea (these last four items combined as “Major physical symptoms”—MPS); and chest pain, arrythmias, headache, sleep disturbances, anxiety, depression (not pre-existing or worsened), memory dysfunction, brain fog, (these last four items combined as “Major Neuropsychological Symptoms”—MNS), paresthesias, muscle pain, joint pain, and sensorial deficit. For each symptom or combination of symptoms, there was a distinction between resolved and ongoing status. Details on ongoing therapies acting on the hemostatic system at the date of follow up were collected.

A blood sample was collected during the same follow-up visit. The coagulation profile was assessed both through standard laboratory analysis and a point-of-care analysis by ClotPro (Haemonetics, Boston, MA, USA).

Standard laboratory assessment included coagulation tests (activated Partial Thrombin Time, aPTT, seconds; international normalized ratio, INR; fibrinogen, mg/dL; D-dimer, ng/mL; antithrombin activity, %) and platelet count (cells/µL).

ClotPro is a CE-marked semi-automatic viscoelastic in vitro point-of-care device featuring the Active Tip™ technology with ready-to-use tips pre-filled with reagents and working with 340 µL citrated blood. The system is composed by a stationary pin and a rotating cup and a typical viscoelastic curve is produced as the clot develops. Samples were analyzed within 30 min of blood draw. Four kind of tests were performed: EXtest (tissue factor activated), INtest (ellagic acid activated), FIBtest (functional fibrinogen), and TPAtest (r-tPA induced fibrinolysis with extrinsic pathway activation). For the EXtest, INtest, and FIBtest, the following parameters were considered: CT (coagulation time, seconds), MCF (maximum clot firmness, mm) and ML (maximum lysis, %). For the TPA test, the LT (lysis time, time required to dissolve 50% of the MCF of the clot once MCF is reached, seconds) and ML (%) were included in the analysis. Data were collected in an electronic platform (Research Electronic Data Capture–RedCAP).

### 4.3. Statistical Analysis

Data are shown as number (percentage), mean (standard deviation), or median (interquartile range), as appropriate. The differences between categorical variables were assessed using a Pearson’s chi square, while differences in continuous variables were explored with a student’s t test (normally distributed variables) or a non-parametric test (non-normally distributed variables). The association between the duration of the follow-up (months) and the persistence of a procoagulant profile was assessed using polynomial function regressions (best fit based on R^2^ value) with a 95% confidence interval. For the statistical calculations and graphical support, data were exported from RedCAP into statistical packages (SPSS 20.0, IBM, Chicago, IL, USA and GraphPad 9.2.0, GraphPad Software, Inc., San Diego, CA, USA). For all tests, a *p* value < 0.05 was considered significant.

## Figures and Tables

**Table 1:** Patient population (N = 102) details during the acute phase of the disease and at follow-up.

ACUTE PHASE	
Item	Value
Age at hospital admission (years)	63.8 (13.1)
Gender male	67 (65.7%)
Weight (kgs)	80.4 (17.8)
Body mass index (kg/m^2^)	27.6 (5.4)
Hospital stay (days)	14 (10–23)
Unit of admission	
Ward	97 (95.1%)
Intensive Care Unit	5 (4.9%)
Vaccination (at least 2 doses)	15 (14.7%)
Obesity	24 (23.5%)
Arterial hypertension	44 (43.1%)
Diabetes	15 (14.7%)
Coronaropathy	12 (11.8%)
Heart failure	6 (5.9%)
Smoking habit	
No	50 (49%)
Previous	48 (47.1%)
Ongoing	4 (3.9%)
Atrial fibrillation	7 (6.9%)
Active cancer previous 5 years	9 (8.8%)
Chronic obstructive pulmonary disease	5 (4.9%)
Chronic kidney failure	6 (5.9%)
Previous cerebrovascular accident	3 (2.9%)
Anxiety	16 (15.7%)
Depression	12 (11.8%)
Chronic liver failure	3 (2.9%)
Therapy	
Beta-blockers	17 (16.7%)
Angiotensin converting enzyme inhibitors	13 (12.7%)
Sartans	8 (7.8%)
Warfarin	2 (2%)
Direct oral anticoagulants	3 (2.9%)
Antiplatelet agents	18 (17.6%)
Calcium antagonists	14 (13.7%)
Statins	15 (14.7%)
Laboratory exams (acute phase)	
Peak fibrinogen (mg/dL)	611 (152)
Peak D-dimer (ng/mL)	3118 (7620)
Peak platelet count (×1000 cells/µL)	329 (110)
Nadir platelet count (×1000 cells/µL)	180 (68)
Nadir antithrombin (%)	100 (15)
Procoagulant state at any time	99 (97.1%)
**FOLLOW-UP**	
**Item**	**Value**
Follow-up time (months)	17 (13–18.5)
Persistent major physical symptoms	60 (58.8%)
Fatigue	50 (49%)
Dyspnea	44 (43.1%)
Cough	13 (12.7%)
Fever	2 (2%)
Persistent major neuropsychological symptoms	44 (43.1%)
Anxiety	23 (22.5%)
Depression	21 (20.6%)
Memory dysfunction	34 (33.3%)
Brain fog	10 (9.8%)
Anticoagulant/antiplatelet therapy	
Dual antiplatelet therapy	4 (3.9%)
Warfarin	1 (1%)
Direct oral anticoagulants	7 (6.9%)
Procoagulant state after hospital discharge	38 (37.3%)

Data are mean (standard deviation), median (interquartile range) or number (%).

**Table 2:** Hemostatic profile at follow up according to the presence of persistent major physical or neuropsychological symptoms. N = 102.

Item	MPS	No MPS	Mean Difference	*p*	MNS	No MNS	Mean Difference	*p*
	N = 60	N = 42	(95% C.I.)		N = 44	N = 58	(95% C.I.)	
INR	1.09 (0.09)	1.1 (0.24)	0.03 (−0.05 to 0.09)	0.578	1.09 (0.09)	1.1 (0.2)	0.01 (−0.06 to 0.07)	0.867
aPTT (s)	28.8 (5.0)	28.4 (4.4)	0.96 (−2.4 to 1.4)	0.607	28.4 (3.8)	28.9 (5.4)	0.45 (−1.4 to 2.3)	0.641
Fibrinogen (mg/dL)	313 (68)	283 (58)	−31 (−56 to −4.7)	0.021	299 (70)	302 (64)	2.7 (−24 to 29)	0.836
D-dimer (ng/mL)	564 (577)	476 (645)	−88 (−331–154)	0.473	544 (479)	514 (690)	−30 (−272–211)	0.806
Platelet count (×1000 cells/µL)	216 (65)	222 (62)	6.2 (−19 to 32)	0.628	218 (76)	219 (52)	0.9 (−24 to 26)	0.943
Antithrombin (%)	90 (13.4)	94 (10.5)	4.8 (−0.5 to 10)	0.073	92 (12)	91 (13)	−1.4 (−6.7 to 3.9)	0.603
EXtest CT (s)	69 (14)	69 (14)	−0.19 (−5.8 to 5.4)	0.947	68 (13)	69 (14)	1.6 (−3.9 to 7.1)	0.567
EXtest MCF (mm)	61 (49)	60 (3.4)	−1.4 (−2.8 to 0.2)	0.090	62 (4.2)	60 (3.4)	0.8 (−2.7 to 0.3)	0.127
EXtest maximum lysis (%)	4.5 (2.3)	5.9 (2.6)	1.42 (0.44 to 2.39)	0.005	5.0 (2.4)	5.2 (2.6)	0.16 (−0.85 to 1.18)	0.753
INtest CT	157 (30)	158 (18)	1.16 (−9.1 to 11.4)	0.823	152 (27)	162 (24)	5.1 (−0.2 to 20)	0.056
INtest MCF	60 (3.9)	59 (3.3)	−1.7 (−3.1 to −0.2)	0.026	60 (4)	59 (3.4)	0.74 (−2.5 to 0.4)	0.171
INtest maximum lysis (%)	4.1 (2.3)	5.5 (2.5)	1.37 (0.40 to 2.33)	0.006	4.6 (2.5)	4.8 (2.5)	0.50 (−0.81 to 1.18)	0.713
FIBtest MCF	20 (4.9)	18 (3.9)	−1.5 (−3.3 to 0.4)	0.117	20 (4.7)	19 (4.3)	−1.0 (−2.8 to 0.79)	0.265
TPAtest lysis time (s)	227 (47)	209 (36)	−18 (−35 to −0.7)	0.033	232 (47)	210 (39)	−22 (−38 to −5)	0.012
TPAtest maximum lysis (%)	95 (1.3)	95 (1.0)	−0.22 (−0.7 to 0.26)	0.346	95 (1.1)	94.5 (1.2)	−0.7 (−1.1 to −0.22)	0.005

Data are mean (standard deviation). aPTT: activated partial thromboplastin time; C.I.: confidence interval; CT: clotting time; EXtest: extrinsic pathway test; INR: international normalized ratio; INtest: intrinsic pathway test; FIBtest: fibrinogen test; MCF: maximum clot firmness; MNS: major neuropsychological symptoms; MPS: major physical symptoms; TPA: tissue plasminogen activator.

## Data Availability

The original dataset supporting the findings of this study will be deposited in the public repository, Zenodo, after the publication of the paper, and accessible upon a reasonable request to the corresponding author.
